# Postoperative Pyoderma Gangrenosum in a Laparoscopic Gastrectomy Port Site: A Case Report

**DOI:** 10.14789/jmj.JMJ22-0017-CR

**Published:** 2022-08-15

**Authors:** SUGURU YAMAUCHI, YUJI ANDO, SANAE KAJI, CHEN JUN, HIROKI EGAWA, YUTARO YOSHIMOTO, AKIRA KUBOTA, YUKINORI YUBE, HAJIME ORITA, TETSU FUKUNAGA

**Affiliations:** 1 Department of Esophageal and Gastroenterological Surgery, Juntendo University Hospital, Tokyo, Japan; 1 Department of Esophageal and Gastroenterological Surgery, Juntendo University Hospital, Tokyo, Japan; 2 Department of Coloproctological Surgery, Juntendo University Hospital, Tokyo, Japan; 2 Department of Coloproctological Surgery, Juntendo University Hospital, Tokyo, Japan

**Keywords:** postoperative pyoderma gangrenosum, laparoscopic gastrectomy, surgical site infection

## Abstract

**Background:**

Postoperative pyoderma gangrenosum (PPG) is a rare inflammatory skin disease of unknown etiology characterized by blistering and ulcerative lesions in postoperative wounds. Untreated pyoderma gangrenosum (PG) is potentially life-threatening; therefore, immediate and appropriate treatment is essential. Although PPG and surgical site infection (SSI) present similar clinical findings, they should be differentiated because of their conflicting treatment modalities.

**Case presentation:**

An 82-year-old man with comorbidities of pulmonary tuberculosis, chronic obstructive pulmonary disease, and diabetes underwent laparoscopic gastrectomy for gastric cancer. On postoperative day 6, fever exceeding 39°C, port wound redness, and pain was observed. Laboratory tests revealed severe inflammatory reactions: white blood cell, 42,800/μL and C-reactive protein, 30.2 mg/mL. The patient was diagnosed with SSI and treatment with antibiotics and drainage was started; however, his general and wound conditions also worsened. Therefore, he was diagnosed with PG because painful skin findings were exacerbated by external stimuli and no significant bacteria were detected in the culture test. Treatment with oral prednisolone was started, which significantly improved his skin and inflammatory conditions.

**Conclusion:**

We managed a rare case of PPG that occurred in a port wound after laparoscopic gastrectomy. If atypical clinical findings of postoperative SSI are observed, general surgeons should recognize and consider PPG as a differential diagnosis.

## Background

Pyoderma gangrenosum (PG) is an uncommon, rare ulcerative skin disease characterized by a rapidly enlarging necrotic ulceration with an undermined border and surrounded by erythema^1, 2)^. Untreated PG is potentially life-threatening; therefore, immediate and appropriate treatment is essential. Postoperative pyoderma gangrenosum (PPG) is also designed as postsurgical or pathergic PG and is closely associated with superficial granulomatous pyoderma^3)^. Although PPG and surgical site infection (SSI) presents similar clinical findings, they should be differentiated because of their conflicting treatment modalities.

## Case presentation

**Figure 1:**
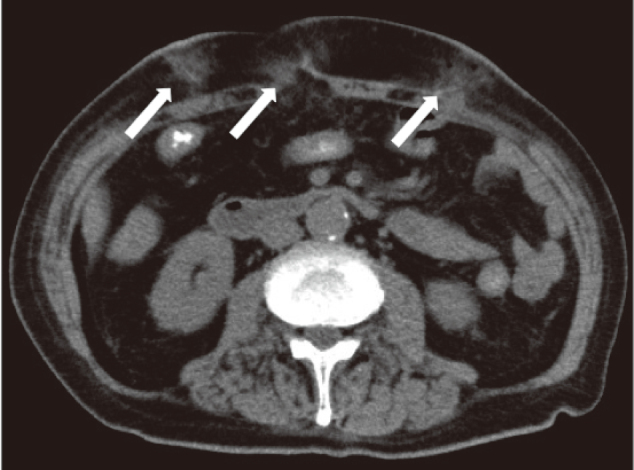
Computed tomography findings No postoperative intra-abdominal events but fluid retention in the subcutaneous tissue of the port site.

**Figure 2:**
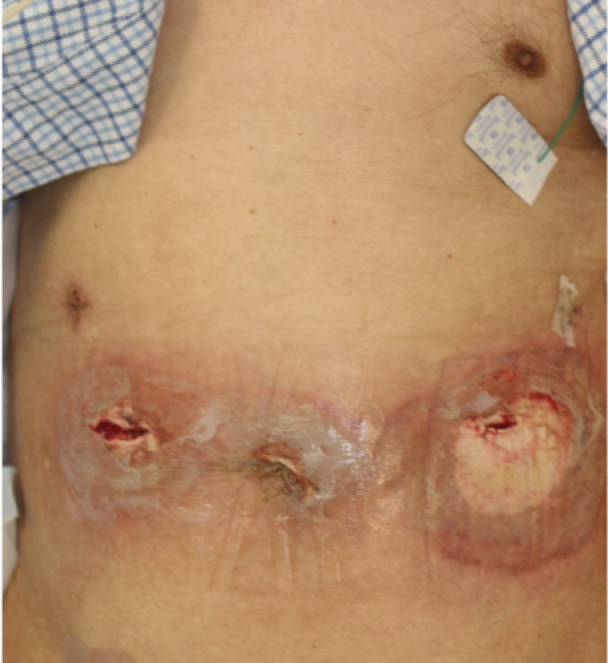
Skin findings on the sixth postoperative day after drainage treatment Blisters and erosions were formed around it, and some necrotic findings with ulcers were also observed.

**Figure 3:**
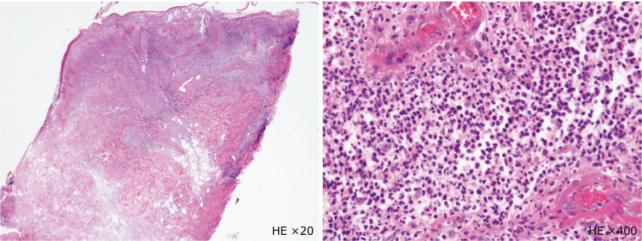
Pathological findings by skin biopsy High degree of neutrophil infiltration from the epidermis to the dermis and multilocular cysts, consistent with PG.

**Figure 4:**
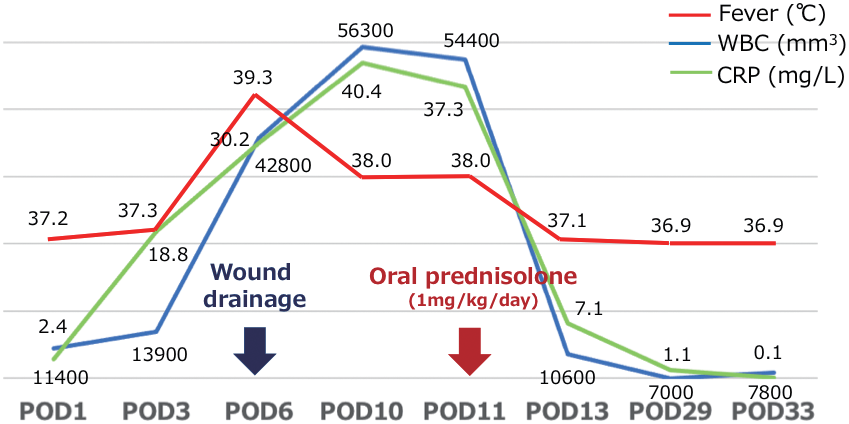
Changes in inflammatory findings Inflammatory findings (fever, white blood cell, C reactive protein), which were aggravated by wound drainage, improved quickly after the start of treatment as PPG.

**Figure 5:**
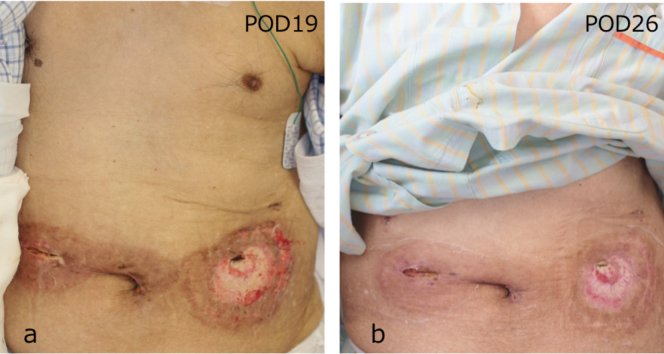
Changes in skin findings In addition to oral prednisolone, wound treatment by maintaining a moist environment that avoids external stimuli resulted in significantly improvement.

An 82-year-old man with comorbidities of pulmonary tuberculosis, chronic obstructive pulmonary disease, and diabetes underwent upper gastrointestinal endoscopy performed by close examination of the black stool and was, therefore, diagnosed with gastric cancer. Laparoscopic distal gastrectomy was performed. The ports were positioned in a reverse trapezoid shape, as commonly practiced. The umbilical port was 12 mm in size. The port below the right hypochondrium was 5 mm in size, and the other three ports were 12 mm. The operation time and bleeding volume were 175 min and 35 ml, respectively, and no intraoperative complications occurred. Until postoperative day 5, only low-grade fever and prolonged inflammation were observed, but without specific clinical symptoms. On postoperative day 6, fever exceeding 39°C, port wound redness, and pain was observed. Laboratory tests revealed severe inflammatory reactions: white blood cell, 42,800/μL and C-reactive protein, 30.2 mg/mL. Computed tomography showed no postoperative intra-abdominal events but fluid retention in the subcutaneous tissue of the port site (Figure 1). He was diagnosed with SSI; therefore, the umbilical, right, and left latero-abdominal port wounds were opened and drained, and a Penrose drain^®^ was placed. After administering broad-spectrum antibiotics and continuing the lavage treatment, the wound condition worsened, blisters and erosions were formed around it, and some necrotic findings with ulcers were also observed (Figure 2). Furthermore, the inflammatory reaction became even more severe in the laboratory test. The course did not match that of the typical postoperative SSI; therefore, we consulted with a dermatologist. The patient was diagnosed with PPG because of painful skin findings exacerbated by external stimuli and the absence of bacteria in the culture test. Skin biopsy also showed a high degree of neutrophil infiltration from the epidermis to the dermis and multilocular cysts, consistent with PG (Figure 3). The treatment with oral prednisolone of 50 mg/day was started, which significantly improved skin and inflammatory findings (Figure 4, Figure 5a, 5b). Written informed consent was obtained from the patient for publication of this case report and any accompanying images.

## Discussion

We managed a rare case of PPG occurring in a port wound after laparoscopic gastrectomy and required differentiation from SSI. Although the incidence of PPG is rare, most reports of PPG to date have come from dermatologists. Although very infrequent, it is one of the diseases that general surgeons should anticipate in differentiating atypical SSI.

PG is a neutrophilic dermatosis typically presenting as a small pustule, surrounded by a halo of inflammation that rapidly becomes painful ulceration with undermined wound edges and violaceous borders^4, 5)^. The cause of PG remains unknown, and its incidence is approximately 3-10 per million per year^6, 7)^. PG is diagnosed by excluding other similar entities caused by infection, vasculopathies, neoplasms, and various inflammatory conditions^8)^. Inflammatory bowel disease, rheumatologic disorders, and hematologic malignancies are comorbid conditions frequently associated PG^9)^. Untreated PG is potentially life-threatening, and patients with PG have a three-fold increased risk of death compared to the general population; therefore, immediate and appropriate treatment is essential^10-12)^. Aside from mortality, PG may cause pain and adversely impact the quality of life, predispose to secondary infection, disfiguring scarring, and recur^5, 7)^.

PPG is also classified as postsurgical or pathergic and is closely associated with superficial granulomatous pyoderma^3)^. Systemic disease is reported to occur in 50%-78% of patients with PG, whereas a low prevalence of the systemic disease has been reported in PPG^6, 10, 13-15)^. Suggesting that the coexistence of systemic diseases that are typical of PG may not be important in suspecting PPG. No comorbidity of systemic diseases related to PG was observed in this case. PPG commonly occurs in the breast, abdomen, and lower legs, in that order, at an average age of 50 years, and often occurs about 1 week postoperatively^15)^.

The common theme of all PPG literature is that the condition is almost always misdiagnosed as an SSI^15-18)^. Stanislav et al. reported that 73% of patients with PPG were initially misdiagnosed as SSI and eventually treated^15)^. Because fever, wound pain, and increased inflammatory response in blood tests and wound changes are also characteristic clinical findings of SSI; thus, the rarity of PPG can result in a false diagnosis. Currently, laparoscopic and robotic surgery have been introduced and widely performed in gastrectomy for gastric cancer due to their low invasiveness and cosmetic outcomes. The occurrence of SSIs is associated with an excess postoperative hospital stay, decreased quality of life, increased treatment costs, and increased mortality^19)^. Although the standard of care for SSI is indisputably antibacterial therapy and appropriate debridement, a paradoxical relationship between SSI and PPG treatment must be considered. Our case was also regarded as SSI and treated with antibiotics, open drainage, Penrose drain^®^ placed, and wound lavage; however, the wound condition wound severely deteriorated. One of the important points is that drainage, considered to be effective for wound infections, can worsen the wound condition in PPG^15, 20, 21)^. Furthermore, no bacteria were detected in the wound culture test in PPG, and histological examination only shows nonspecific inflammatory findings, which will be useful in ruling out SSI. In the present case, only three of the five port wounds developed PG. The reason for this seems that all three port wounds were 12-mm ports, which are frequently manipulated intraoperatively, and the laparoscopy and forceps manipulations can easily cause strong physical irritation to the wounds, which is possibly contributed to the development of PG.

The treatment for PPG is generally the same as PG; corticosteroid and cyclosporine therapies are supported best by the literature^11, 22)^. Zuo et al. reported that the majority of patients were treated with oral prednisolone (0.5-1.5 mg/kg/day) or intravenous methylprednisolone (0.5-1.0 mg/kg/day) combined with/without cyclosporine^18)^. If the patient is resistant to these treatments, other treatments such as mycophenolate mofetil, infliximab, tacrolimus, or plasmapheresis may be considered^5, 21)^. Moreover, steroid and immunosuppressive therapies for the treatment of PPG are predisposed to wound infections and can exacerbate existing infections; therefore, they should be used cautiously^5, 21, 23)^. As for the treatment of the wound itself, as mentioned earlier, drainage should not be performed as it will worsen the condition. Maintaining a moist environment after washing with saline is important to promote proper wound healing^24)^. Consistent with this case, systemic symptoms, laboratory findings, and wound condition dramatically improved with 50 mg (1 mg/kg/day) of oral prednisolone and wound care that avoided external stimuli as much as possible.

Although PPG is a rare disease, general surgeons should consider PPG as one of the differential diagnoses and treatments when managing an untypical course of SSI. Postoperative wound abnormalities that are resistant to the standard treatment should be referred to a dermatologist at an early stage of treatment, if possible, as the addition of expert judgment can lead to earlier diagnosis and treatment.

## Conclusion

We managed a rare case of PPG that occurred in a port wound after laparoscopic gastrectomy. If atypical clinical findings of postoperative SSI are observed, general surgeons should recognize and treat PPG as a possible differential diagnosis.

## Funding

No funding was received for this report.

## Author contributions

SY, YA and KS drafted the manuscript. KS, YY and AK performed surgery. CJ, HE and YY contributed to patient care. TF, HO gave final approval of the manuscript. All authors read and approved the final manuscript.

## Conflicts of interest statement

The authors declare that they have no conflicts of interests.
